# Photocatalytic Carbon Dioxide Conversion by Structurally and Materially Modified Titanium Dioxide Nanostructures

**DOI:** 10.3390/ijms23158143

**Published:** 2022-07-24

**Authors:** Tarek Fawzi, Sanju Rani, Somnath C. Roy, Hyeonseok Lee

**Affiliations:** 1 Department of Photonics, National Sun Yat-sen University, No. 70, Lien-Hai Rd, Kaohsiung 80424, Taiwan; t@g-mail.nsysu.edu.tw or; 2 Department of Physics, SRM Institute of Science and Technology, Ramapuram Campus, Chennai 600089, Tamil Nadu, India; sanjur@srmist.edu.in; 3 Semiconducting Oxide Materials, Nanostructures and Tailored Heterojunction (SOMNaTH) Lab, Functional Oxides Research Group (FORG) and 2D Materials and Innovation Centre, Department of Physics, IIT Madras, Chennai 600036, Tamil Nadu, India; somnath@iitm.ac.in

**Keywords:** photocatalysis, CO_2_ reduction, TiO_2_, nanostructures and hydrogenation

## Abstract

TiO_2_ has aroused considerable attentions as a promising photocatalytic material for decades due to its superior material properties in several fields such as energy and environment. However, the main dilemmas are its wide bandgap (3–3.2 eV), that restricts the light absorption in limited light wavelength region, and the comparatively high charge carrier recombination rate of TiO_2_, is a hurdle for efficient photocatalytic CO_2_ conversion. To tackle these problems, lots of researches have been implemented relating to structural and material modification to improve their material, optical, and electrical properties for more efficient photocatalytic CO_2_ conversion. Recent studies illustrate that crystal facet engineering could broaden the performance of the photocatalysts. As same as for nanostructures which have advantages such as improved light absorption, high surface area, directional charge transport, and efficient charge separation. Moreover, strategies such as doping, junction formation, and hydrogenation have resulted in a promoted photocatalytic performance. Such strategies can markedly change the electronic structure that lies behind the enhancement of the solar spectrum harnessing. In this review, we summarize the works that have been carried out for the enhancement of photocatalytic CO_2_ conversion by material and structural modification of TiO_2_ and TiO_2_-based photocatalytic system. Moreover, we discuss several strategies for synthesis and design of TiO_2_ photocatalysts for efficient CO_2_ conversion by nanostructure, structure design of photocatalysts, and material modification.

## 1. Introduction

Plants preserve carbon cycle in nature via photosynthesis process as they convert CO_2_ into carbohydrates, but several activities such as industrialization, urbanization, deforestation and excessive consumption of fossil fuels affected this cycle considerably [1]. The accumulation of CO_2_ in the atmosphere, beyond the capacity of the nature to handle, has created severe global warming phenomena that result in climate change. Now, the devastating effects from unusual weather patterns are visible all around us. In the recent years, therefore, CO_2_ capturing and utilization is a focal subject for the scientific community [2,3], as it addresses the energy crisis and global warming without hindering the development plans or urbanization [4]. In particular, recycling of CO_2_ into carbon containing and value-added chemicals regenerates fuel within the present hydrocarbons-based energy infrastructure.

CO_2_ is a linear molecule, with weak electron affinity, chemically stable and the nucleophilic approach at the carbon atom governs its conversion reaction [5]. C=O bond dissociation requires more than 750 kJ/mol of energy [6]. Thermodynamically, this reaction is an uphill one, hence input of energy is required to break C=O bond. In nature, the photosensitizers (e.g., chlorophyll) capture energy from sunlight to use it in the endothermic reactions.

To mimic these natural solar energy conversion, several techniques such as photoelectrochemical (PEC) [7], biochemical [8], photocatalysis [9], radiolysis [10], thermo-catalysis [11], and electrocatalysis [12] have been proposed for CO_2_ conversion into useful chemicals. Photocatalytic (PC) methods has been identified as one of the most suitable approaches to convert CO_2_ into different gaseous (ethane, methane, etc.) and liquid (ethanol, methanol, formate etc.) products under solar light irradiation at ambient temperature and pressure [13]. The most attractive aspect of such approach is the utilization of renewable solar radiation to draw the energy required for driving the catalytic process. In the case of a pure photocatalytic approach (without any electric field), this is called artificial photo-synthesis that imitates the energy cycle of nature [14]. In addition, use of renewable solar energy brings in associated advantages such as environmental compatibility, economic feasibility, and product selectivity [15].

Up to now, several different types of materials such as metal complexes, organic molecules, precious metals, ions, organic molecules, and semiconductors have been explored to enhance the efficiency of photocatalytic processes. Among these materials, semiconductors in general and metal oxides in particular have shown immense potential over the others with regard to stability, toxicity, and feasibility of the fabrication etc., which makes them desirable in photocatalytic applications [16].

The general understanding for an efficient photocatalytic process by semiconductor materials, the electrons and holes in the semiconductor materials should have sufficient energy to ionize the CO_2_ and H_2_O, respectively. In other words, energies of the photogenerated electron and hole (e^−^/h^+^) must be higher than the overpotential of H_2_O/O_2_ (0.82 vs. NHE at pH 7) and lower than the overpotential of CO_2_/HCOOH (−0.61 V vs. NHE at pH 7), respectively [17,18]. Performing photo-assisted redox reactions such as CO_2_ reduction or water oxidation therefore requires a photocatalyst with an optimum bandgap (at least 2.88 eV) [19] so as to generate electrons and holes with sufficient energies. Simultaneously, the photocatalyst must be able to absorb large fraction of the solar spectrum even from visible and near IR light absorption (in addition to UV) [20].

Historically, TiO_2_ is the most extensively investigated semiconductor material for photocatalytic CO_2_ conversion since the successful demonstration of the photoelectrochemical CO_2_ conversion by Inoue et al. in 1979 [21]. Moreover, owing to superior advantages such as material robustness, chemical and thermal stability, nontoxicity, corrosion resistance, and ease of synthesis, TiO_2_-based photocatalytic systems became popular and related applications have been focal target for researchers due to its tremendous potential in different solar driven processes such as water splitting, pollutant degradation and CO_2_ conversion [21,22]. It is well known TiO_2_ has three crystalline phases, anatase, rutile, and brookite possessing the bandgap values in the range of 3.0–3.6 eV. However, the bandgap is relatively large to absorb whole solar spectrum and this limits the light absorption of TiO_2_ only to UV region, which is ~5% of total solar energy available. Moreover, the charge recombination in TiO_2_ is a serious issue and this makes the photocatalytic reaction by TiO_2_ inefficient. Hence, increasing the optical absorption and decreasing the charge recombination of the TiO_2_ are of another prime importance. To overcome these limitations, lots of research has proposed on material modification, low dimensional introduction, and design of photocatalytic structure for TiO_2_ to achieve highly efficient photocatalytic CO_2_ conversion.

Therefore, in this review, we summarize the works that have been carried out for the enhancement of photocatalytic CO_2_ conversion by material and structural modification of TiO_2_ and TiO_2_-based photocatalytic system. We provide several strategies and their details: (1) crystal facet engineering (2) nanostructures (3) junction formation (4) material modification, in particular, by hydrogenation (5) single atom photocatalysts (6) metal-organic framework.

## 2. Strategy I: Crystal Facet Engineering of TiO_2_ Photocatalyst

**Figure 1:**
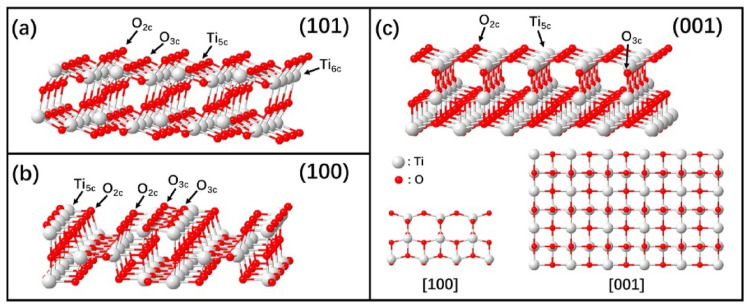
Ball and stick models of anatase TiO_2_ low-index surfaces: (**a**) (101) surface; (**b**) (100) surface, and (**c**) (001) surface (red: oxygen; grey: titanium). (Taken with permission from [28]).

Titanium dioxide is a multifunctional material that has attracted researchers due to its applicability to various fields such as photocatalysis, photovoltaics, and biomedical applications [23,24]. TiO_2_ nanoparticles and nanocrystals have been obtained via different preparation methods such as flame spray pyrolysis, hydrothermal treatment and sol-gel method. In particular, crystallites with selective facets are helpful in catalysis. In addition to wet chemistry approaches, powders produced from flame pyrolysis resulted in nanocrystals with thermodynamically stable (101) surface [25,26] and (100)/(010) surface planes, and some reactive (001) facets (Figure 1 shows the different types) [27,28].

While, the size of TiO_2_ nanoparticles differs remarkably depending on the synthesis process [29], the preparation techniques affect the crystal shapes significantly [30]. Choosing a specific synthesis method depends on the required morphology of TiO_2_ material, which further depends on the target application. The methods of synthesis significantly affect the morphology, crystallinity, and phase, which have their influence on the physical and chemical properties. The synthesis strategies are chosen, considering the ability to tailor particle-size, the integration with other structures or phases [31], flexibility of self-assembly [32], possibility of doping with other elements [33,34], also inlaying heteroatoms, heterostructures or quantum dots [35,36] in order to manipulate the electronic and optical properties.

Anatase is the most investigated photoactive polymorph of TiO_2_, with its thermodynamically stable (101) facets (>94%), dominating in consonance with the Wulff construction. The morphology, crystal growth, and facets can be controlled by using a shape and growth controller such as HF [28]. In addition, in another approach, a mixture of HF/alcohol has been employed for establishing metastable surfaces reaching 98.7% of (001) and 1.3% of (100) facets [37]. Using F^−^ along with citric acid or hydroxyl acids results in continuous curvature of rutile and anatase, where F^−^ ions play not only the role of a stabilizer for (001) facets growth but also as an etching reagent [38,39]. The morphology of the anatase nanoparticles can be manipulated via the relative concentrations of OH^−^ and F^−^ [40]. Hydroxyl groups boost the isotropic growth, whereas F^−^ eliminates (001) surfaces supporting the TiO_2_ crystals lateral growth. However, an excessive concentration of F^−^ reduces the size of particles significantly as a result of TiOF_2_ formation.

The facet engineering effect was reported by Wang et al. [41]; where they found that the Schottky barrier height of Au/TiO_2_ (101) interface is lower than its counterparts from the interface (001). Which enhanced the electrons transfer from CB of TiO_2_ to Au and significantly enhances the photocatalytic performance in producing CO and CH_4_ in comparison with other samples containing Au/TiO_2_ (001) interfaces. In another study, Dong et al. [42] showed that crystal facets engineering of TiO_2_ loaded with Cr_2_O_3_, (2HF-TiO_2_/0.2Cr_2_O_3_) exhibited 30-fold increment in CO_2_ conversion compared to the TiO_2_ without facet engineering.

**Figure 2:**
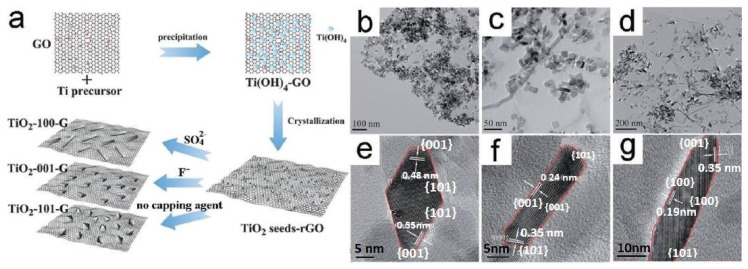
(**a**) Schematic illustrating the synthesis of TiO_2_-graphene nanocomposites with controllable TiO_2_ crystal facets. (**b**–**g**) transmission electron microscopy (TEM) and high-resolution TEM (HRTEM) images of the as-prepared (**b**,**e**) TiO_2_-101-G, (**c**,**f**) TiO_2_-001-G, and (**d**,**g**) TiO_2_-100-G. (Taken with permission from [54]).

Studies and theories illustrated that (101) facets are the most stable facets thermodynamically for anatase TiO_2_, whilst other facets (100) and (001) are active and possess high surface energy [43]. Anatase crystal has an equilibrium shape consisted of slightly truncated tetragonal bipyramid enclosed with two (001) and eight (101) facets [44]. Tetragonal nanorods of anatase crystals consisted of high ratio of lateral (100) facets have been fabricated via immersing alkali titanate nanotubes in basic solution followed with hydrothermal transformation [45]. In addition, Anatase crystals with elongated truncated tetragonal bipyramids that contains high ratio of (100) facets; enclosed via different types of facets (001), (100) and (101) were fabricated via hydrothermal reaction in aqueous HF solution [46]. However, still a dilemma to fabricate tetragonal cuboid of anatase crystals enclosed only with (001) and (100) facets. Keeping in mind that anatase nanosheets were fabricated by using solvothermal route in 1-butanol solvent containing HF; these nanosheets consists of (100) and (001) facets, 1.3% and 98.7, respectively [47,48]. Preparing anatase cuboids and manipulating facets percentage (100) and (001) may help in evaluating facet reactivity throughout the photocatalytic reactions. Hence, it is for crucial significance to synthesize anatase cuboids enclosed by (001) and (100) facets over a wide size range, as in lithium-ion batteries and solar cells where several studies [49,50,51,52] relied on the exposed facets of anatase effect on its optical and electrochemical features to be applied in such applications. Photocatalytic materials can be formed via the integration of multiple components, adjusting the facet of each component. This method enables to boost the photocatalytic performance but, since the facet engineering in the multiple component system, in general, are complicated for its delicate control, the combination of two pre-synthesized components is more preferable for its usage, rather than a new component on the one pre-synthesized material [53]. For example, tailoring the surface facets of TiO_2_ seeds into nanocrystals with graphene oxide can be seen in Figure 2a. capping agent deficiency results in octahedral TiO_2_ nanocrystals enclosed by TiO_2_-101-G. TiO_2_-001-G nanosheets and TiO_2_-100-G nanorods were formed on graphene with F^−^ and $SO_{4}^{2-}$ Semployed as capping agents, as shown in Figure 2b–g).

## 3. Strategy II: Nanostructured TiO_2_

Although TiO_2_ in its bulk form has been investigated for decades, its nanostructured morphologies also have been investigated for photocatalysis and other applications [55], focusing on one dimensional (1D) [56], two dimensional (2D) [57], or three dimensional (3D) structures [58]. Nanostructures or nanomaterials are defined as features where at least one dimension is smaller than 100 nm [59]. However, in a more technically elaborated way, a nanomaterial is defined as where charge carriers are quantum-mechanically confined as evidenced by the consequent modifications in electronic and optical properties. However, even before the electronic or optical properties are investigated, the nanomaterials are characterized by a huge enhancement in the surface to volume which enhances the interaction with the surrounding environment [60]. Each of these nanostructures have their own characteristics in terms of light scattering, aspect ratio, recyclability, major surface area, stability, and directional transfer of photogenerated charges that decreases recombination rate. Synthesis method for 1D nanostructures involves tailored and directional growth, which is achieved either by the structure directing agents or by the use of templates. The directional growth is not required for 2D and 3D morphologies, but the synthesis process may involve multiple steps and highly optimized conditions to obtain structures with minimal defects. In the case of 0D nanostructures such as quantum dots, although there have been studies reported in the literature, the enhancement in band gap caused by the quantum confinement effect reduces the effectiveness of use in photocatalysis. Following chapters provide fundamental features of each nanostructure and its application for CO_2_ conversion.

### 3.1. Zero-Dimensional Nanostructured TiO_2_

The nanoparticles or 0D nanostructures offer a lot of advantages and a high degree of flexibility in terms of use in photocatalysis. In particular, nanoparticles can be used to functionalize various surfaces of materials that help in forming either heterojunctions or modifying light absorption. Further, tailoring the size of the nanoparticles changes the surface area, which, in turn, influence the catalytic reactions. Moreover, when formed into quantum dots, the optical and electronic properties are modified drastically to help in catalytic reactions. These nanostructures are cheap, stable, recyclable, and biocompatible [61]. TiO_2_ nanoparticles with their special morphology and acid-base sites have been useful in many catalytic reactions under mild conditions compared to other metal oxides (e.g., CuO, ZnO, etc.) [62]. Liu et al. [63] constructed 0D nanoparticles/2D CoP nanosheets heterojunction and the results showed improvement in photocatalytic H_2_ evolution rate in comparison to pure TiO_2_. Another study [64] reported that In-doped TiO_2_ nanoparticles improved the photocatalytic activity for CO_2_ reduction where CO was detected and CH_4_ yield increased remarkably. In addition, Wada et al. [65] employed rutile TiO_2_ nanoparticles as a modifier to enhance the charge transfer, where RuRe/TiO_2_/NS-C_3_N_4_ showed capability in converting CO_2_ into CO with high selectivity under visible light (λ > 400). Tseng et al. [66] used sol-gel method in synthesizing Cu/TiO_2_ nanoparticles and measured the photoreduction that showed methanol yield much higher than those resulted from sol-gel TiO_2_ and Degussa P25, as well. Moreover, Pt/TiO_2_ nanoparticles composites yield CH_4_, H_2_ and C_2_H_6_ under visible light irradiation with increment of 3.7 times in comparison with Pt/P25 [67]. Perovskite quantum dots also showed its potential for photocatalytic CO_2_ conversion with TiO_2_ [68,69].

### 3.2. One-Dimensional Nanostructured TiO_2_

**Figure 3:**
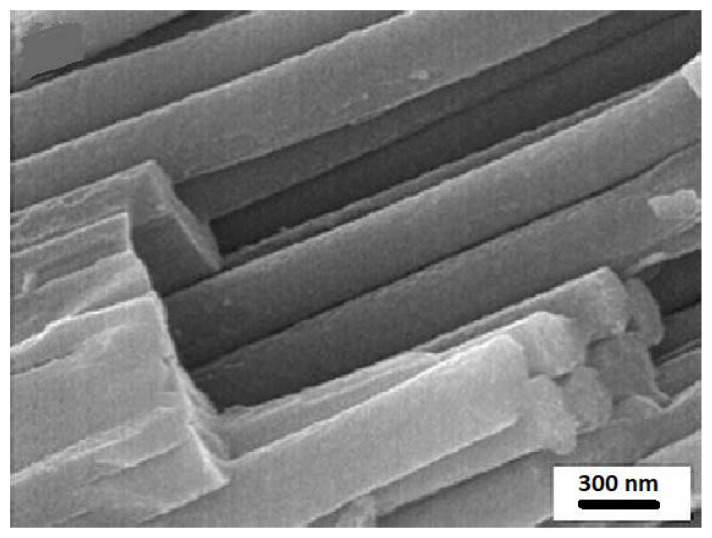
Field emission scanning electron microscopy image of the synthesized TiO_2_ nanorod arrays. (Taken with permission from [79]).

The one-dimensional morphologies such as nanowires, nanorods, nanobelts and nanotubes have several interesting properties including directional charge transport, improved light absorption by high aspect ratio and widen surface area [70]. A lot of research reports further modified properties of nanostructures through doping or decoration of other materials such as graphene derivatives or noble metals to enhance photocatalytic CO_2_ conversion with high aspect ratio [71,72]. The integration of the specific geometry and the high aspect ratio yield dramatical enhancement in charge carrier generation, separation, and transport which boost the conversion efficiency [73]. For instance, TiO_2_ nanorods (TNRs) have a single-crystalline structure and small boundary resistance [74], that reduces the impact from grain boundaries and supplies fast electron transportation [75,76]. It was reported that TNRs as shown in Figure 3 shows higher photocatalytic activity than the nanoparticles because of the increment in active sites and the influence of the crystal plane [77]. Moreover, the comparison between TiO_2_ nanorods and the counterparts of nanoparticles showed that the recombination rate of the nanorods are lower that enhanced the photocatalyst photocatalytic activity [78].

Wang et al. [80] fabricated a one-dimensional TiO_2_ single crystal with ultrafine Pt nanoparticles (0.5–2.0 nm) by versatile gas-phase deposition. This film showed extremely high efficiency in CO_2_ photoreduction with selective formation of methane compared to pristine P25. In addition, TiO_2_ nanotubes fabricated by Ping et al. [81] reduced CO_2_ with H_2_ into methanol and ethanol with photocatalytic performance higher than that of TiO_2_ nanoparticles. Another study used microwave solvothermal approach in deposition of Pt nanoparticles on TiO_2_ nanotubes (TNT). This composite promoted the photocatalytic conversion of CO_2_ with water into methane [82]. TiO_2_ 1D nanostructured by alkaline hydrothermal method also exhibited its promising CO_2_ conversion performance via the heterostructure formation with some materials such as Nb_2_O_5_, CNM and Bi_2_S_3_ under visible light irradiation [83]. The results showed improved efficiency attributed to the enhanced light absorption and the charge separation [83]. The band edges of the aforementioned materials embedded well over the band edge of TiO_2_; hence, applying light irradiation excites electrons, which, in turn, flow to the conduction band of the TiO_2_ and react with the absorbed CO_2_ species. Although deposition of these materials reduces the surface area and CO_2_ adsorption of the TNT, enhancing charge transfer kinetics brought advantage over the reduction of surface area. In addition, the 1D structures showed excellent performances, combining with various techniques and materials: for examples, TiO_2_ nanoflower films modified with Cu, depositing CdS, grafting CoOx nanoparticles on TNTs with defects via hydrogenation through the heterostructure by N_2_/H_2_ annealing [84], TiO_2_ nanowires (TNWs) loaded with noble metal nanoparticles such as Au [85] or Ag [86], TNT with electrodeposited Ag nanoparticles [87], TNW with Pd nanoparticles [88], TiO_2_ nanobelts (TNB) forming heterostructures with ZnFe_2_O_4_ nanoparticles [89], and TNT covered with rGO sheets with embedded TiO_2_ nanoparticles [90]. All the techniques and materials implemented onto 1D TiO_2_ nanostructures enhanced the photocatalytic performance by improving the product (CO, CH_4_, CH_3_OH, CH, CF) yield under light irradiation because of all or some of these factors: (i) increasing active surface area, (ii) increasing CO_2_ adsorption on the surface (iii) trapping electrons via oxygen defects (iv) enhancing charge separation.

### 3.3. Two-Dimensional Nanostructured TiO_2_

Ultrathin two-dimensional nanomaterials possess sheet-like structure with a thickness of few atoms (less than 5 nm), their widths are larger than several hundred nanometers [91,92]. Their superb physical and chemical properties have led significant attention for diverse lateral structured applications [93]. In comparison with 0D and 1D, 2D nanomaterials have extraordinary advantages; support them with optimistic potential for photocatalytic applications.

- First and foremost, 2D materials possess larger surface-to-volume ratio over their bulk counterparts [94]. Hence, 2D materials have more active sites on their surface that can enhance their photocatalytic performance significantly.
- Second, their atomic thickness benefits mass transport and light energy harvest [93]. The ultrathin structure minimizes the distance of the charge migration from the bulk to the surface, decreasing carrier recombination and enhancing the photocatalytic activity [95].
- Third, the high fraction of coordinated unsaturated centers can work as active centers and interact with the substrate intimately [96].

**Figure 4:**
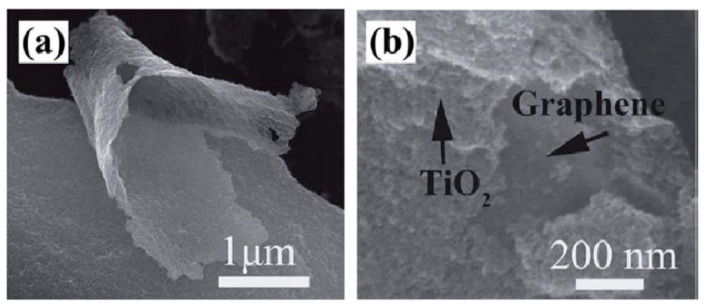
SEM image of 2D nanosheet sandwich-like graphene/TiO_2_. (**a**) The typical G2-TiO_2_ with remarkable structural flexibility. (**b**) The TiO_2_ nanoparticles intimate contact with graphene. (Taken with permission from [98]).

Hence, they perform stellar platforms to prepare multicomponent photocatalysts. The aforementioned extraordinary properties provide diversified number of opportunities with high activity and selectivity for CO_2_ reduction. In particular, synthesizing fine-tuned and strong photocatalysts that fulfil the requirements of CO_2_ reduction applications [97]. These properties encouraged Tu et al. to fabricate 2D sandwich-like hybrid nanosheet out of graphene and TiO_2_ in Figure 4, where TiO_2_ nanoparticles uniformly were loaded onto graphene nanosheet to prevent their breakdown and restacking.

2D structures provide large active surface area, enhanced surface adsorption, enhanced interfacial charge transfer, and selectivity caused by the band edge alignment. Zhou et al. loaded with TiO_2_ nanoparticles onto layered nanosheets of g-C_3_N for CO_2_ conversion [99]. Urea served as a source of N doping as well as g-C_3_N_4_ precursor. Low urea formed an N-doped TiO_2_ and resulted in CH_4_ during the photocatalytic CO_2_ reduction while high urea formed composite of g-C_3_N_4_ and N-TiO_2_ that produced CO. The reason behind the selectivity of products is alignment of band edges with respect to the redox potentials of the possible products. The photocatalytic yield increased due to average surface area, enhanced light absorption, promoted charge transfer and well-aligned band edges with respect of product redox potentials. Likewise, TiO_2_-g-C_3_N_4_ nanosheets heterostructure (TNS-CNN) has been synthesized via in situ pyrolysis approach [100]. TNS-CNN used H_2_O and H_2_ as reducing agents in CO_2_ conversion process where its CO yield was very high compared to pristine TNS. This performance is attributed to the surface area increment, enhanced charge transfer kinetics, role played by H_2_ and light absorption. Ultrathin TiO_2_ nanosheets also play an essential role for efficient photocatalytic CO_2_ conversion, when prepared from the lamella structure of TiO_2_-Octylamine [101]. The conversion efficiency is a result of several factors such as increasing CO_2_ adsorption sites, enormous increasing in surface area, which in turn, increased the light absorption. Moreover, the fluorescence lifespan of the generated charges into the ultrathin TiO_2_ nanosheets is higher when compared to their counterparts in bulk material. Thus, these ultrathin nanosheets provide efficient charge separation within its 2D channels. In another report, TiO_2_ ultrathin nanosheets (TiO_2_-U) were synthesized by hydrothermal method followed by photochemical deposition of Pt nanoparticles [102]. Moreover, an interesting study suggested growing the photocatalytic material onto a 2D conductive substrate. Recently, Ti_3_C_2_ MXenes (TT) has been synthesized and covered with TiO_2_ nanoparticles [103]. Upon calcinating TT at 550 °C, the TiO_2_ nanoparticles formed at the edges and the surfaces of TT layers which improves the surface area by making it rougher. Applying higher temperature than 550 °C decreased the photocatalytic performance due to the decreased proportion of the conductive TT. Hence, TT offers an efficient charge separation that improves the performance and the surface area contributed significantly in providing more reactive sites for CO_2_ adsorption for conversion process, as well. Another report described the 2D nanostructure of Bi_2_WO_6_-TiO_2_ bi-nanosheet (BT) for CO_2_ conversion into CH_4_ and CO [104]. This report provided an approach concerning carbonaceous intermediates or surface species in the value-added chemicals generation. It was found that BT resulted in improved CO and CH_4_ yield compared to pristine material due to the enhanced charge transfer and the Z-scheme mechanism.

**Figure 5:**
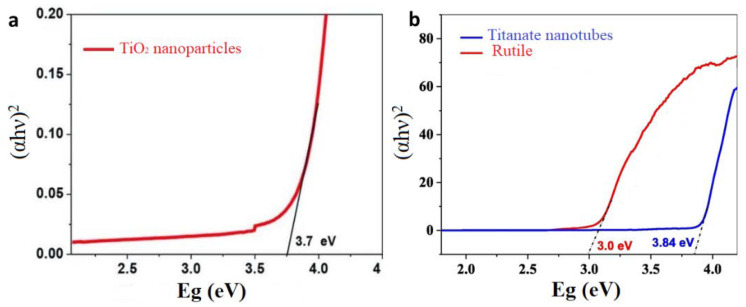
Tauc plots of (**a**) TiO_2_ nanoparticles (**b**) Titanate nanotubes and rutile.

As known, TiO_2_ has three common polymorphs anatase, brookite, and rutile. Brookite is the least used one while rutile is the most common one. It is well known that TiO_2_ has a wide bandgap of 3.0 eV for rutile. In addition to this, it was reported that the bandgap of anatase is about 3.2 eV, while that of the brookite is anywhere between 3.0–3.6 eV. Anatase nanoparticles with grain size (5–10 nm) shows a blue shift in the absorption edge of 10 nm, and their bandgap is about 3.3–3.4 eV in comparison with the commercial sample with a crystal size 39 nm which bandgap is about 3.2 eV [105]. Another study reported that the bandgap of pure TiO_2_ nanoparticles is 3.7–3.9 eV, as shown in Figure 5a [106].

Studies proved that as the material shrinks to the nanometer scale, the bandgap starts to increase due to the quantum size effect [107]. Hence, TiO_2_ nanowires (TNWs) have higher bandgap compared to the bulk materials, for instance growing the TNWs along with (001) direction results in a blue shift and this shift depends on the size of the TNRs [107]. In addition, the bandgap of titanate nanotubes is almost 3.84 eV ascribed to quantum confinement effect, shown in Figure 5b [60]. Moreover, in a comparison between TiO_2_ nanosheets (TNSs) and bulk materials, the spectrum of the TNSs showed significant blue-shift that indicates increasing in the bandgap [108].

## 4. Strategy III: Formation of the Junction with TiO_2_

Photocatalysis CO_2_ conversion is closely related to the management of charge carriers, namely, electrons and holes, that are necessary for CO_2_ reduction and water oxidation, mainly governed by photon absorption, photocarrier generation, and charge separation and transfer. These reactions rely on several factors: bandgap, E_g_ of photocatalyst, photo carrier generation rate, charge transfer kinetics, and recombination rate. Photocarrier generation rate depends on the conditions of the irradiation and the optical absorption properties of photocatalysts while the charge transfer depends on the reaction occurrence whether it takes place at the surface or within photocatalysts. The recombination can be minimized by improving the crystallinity, increasing transfer kinetics, or the control of the crystallite size [109]. Highly crystallized material minimizes the presence of impurities, surface or bulk defects. Crystallite size control stimulates the point defects of particles that trap e^−^ or h^+^ which in turn delays the recombination for a few micro or nanoseconds [95]. In addition, the reaction efficiency can be enhanced by increasing the adsorption capability of the surface [110].

To achieve these improvement and enhancement in properties and performance of TiO_2_ photocatalysts, we can make an effective junction with other materials. As mentioned, since TiO_2_ has disadvantages for efficient light absorption and charge transfer, making a junction with efficient light absorbers or/and charge transporting materials would be beneficial for the enhancement of the performance. The subchapters below provide the features and examples of several junctions that can be made with TiO_2_.

### 4.1. A Semiconductor-Semiconductor Heterojunction

The most typical semiconductor-semiconductor heterojunction can be formed in *p-n* junction. The *p-n* junction, where an *n*-type and a *p*-type semiconductor are in intimate contact (for charge transport across the interface), has several advantages in photocatalysis. When two such semiconductors make a junction, a depletion layer is formed at the interface and internal electric field is established at the interface due to the relative positions of the Fermi levels. This built-in electric field at this junction helps in separating the photogenerated electron-hole pairs thus minimizing the recombination. Further, the junction can be designed in such a way that the light absorption range of one semiconductor can be extended by choosing the other semiconductor with a smaller band gap. Therefore, two fundamental challenges, light absorption and charge carrier separation, in TiO_2_-based photocatalysts *n* photocatalysis- can be tackled by creating *p-n* junction with materials that have suitable band gaps and intimate energy band structure [111].

Moreover, non-*p-n* junction, that is, *n-n* junction between the materials also helps in photocatalysis in a similar manner. In such a heterostructure, the band edge offsets at conduction and valance bands endow a driving force for charge transfer and separation and, with a proper selection of materials, enhanced light absorption can be expected. Such a configuration, called a type II heterojunction is highly useful in photocatalysis [112].

These semiconductor-semiconductor heterojunction structure has been demonstrated in lots of work in the field of photocatalysts. Guo et al. [113] reported that Bi_2_WO_6_/TiO_2_ heterojunction photocatalysts showed strong adsorption ability and improved visible light photocatalytic activities. Shang et al. [114] also studied Bi_2_WO_6_/TiO_2_ photocatalytic activity as it demonstrated enhanced photocatalytic activity by 8 orders compared to the bare Bi_2_WO_6_. MgO^−^ covered TNT via alkaline hydrothermal reaction also demonstrated for CO_2_ conversion [115]. This heterojunction enhanced the CO_2_ photoreduction as indicated by higher amounts of CO and CH_4_ compared to that from sole TNT photocatalysts. This enhancement is attributed to the chemisorption of CO_2_ and subsequent conversion into MgCO_3_ which is more reactive than the linear molecules of CO_2_. Li et al. [83] fabricated TNT via hydrothermal reaction followed by constructing CdS/TiO_2_ and Bi_2_S_3_/TiO_2_, the photocatalytic performance of both of them enhanced CO_2_ reduction into CH_3_OH under visible light irradiation. In addition, Li et al. [115] presented a study showing the development of MgO/TNTs films in CO_2_ photoreduction activity into methane in comparison with TiO_2_ films where MgO played a crucial role in CO_2_ methanization.

### 4.2. Semiconductor-Metal Heterojunction

TiO_2_ photocatalysts forms a junction with various metal materials to enhance the photocatalytic performance. A metal in contact with a semiconductor creates Schottky or ohmic contact and these contacts influence on electric field or charge concentration distribution at interface. As in the semiconductor-semiconductor junction above, the metal materials can affect the electrical properties of the photocatalytic structures for charge separation and transfer of the semiconductor materials forming a junction together. In addition, some special metals such as Ag, Au, or Cu help in extending light absorption with suitable plasmonic effect when they make a junction with TiO_2_ [116,117,118,119]. Further, metals with suitable surface energies also help in enhancing the adsorption of the gaseous/liquid species under catalytic reaction [18]. Wang et al. [120] successfully fabricated Au/TiO_2_ heterojunction that resulted in reduction products CH_4_ and CO, with 80% CH_4_ selectivity. Saraev et al. [121] modified TiO_2_ with Pt and Cu/CuO_x_ and reported that this design is an efficient photocatalyst for CO_2_ conversion as it shifted the working range to the visible light and produced CH_4_. Another study reported that Au0.25Pt0.75/TiO_2_ nanofiber showed higher activity of CO_2_ photoreduction into CH_4_ under UV-vis irradiation [122]. In addition, Mankidy et al. [123] reported that Ag-Pt bimetallic and core sell Ag@SiO_2_ onto TiO_2_ showed a significant development in the photoreduction of CO_2_ with H_2_O into CH_4_.

### 4.3. Semiconductor-Carbon Heterojunction

Similarly, to the metal-semiconductor junctions, carbon materials are also used to form heterostructures with photoactive semiconductors. Several carbon materials such as activated carbon, graphene, and graphene oxide have been employed in heterojunctions preparation. These materials demonstrate high surface area that improves molecules adsorption and the photocatalysis performance of the material [124]. The porous morphology demonstrated by carbon materials adsorbs gaseous species for catalytic reactions. Further, graphene and carbon nanotube (CNT) have a metallic structure and forms a Schottky barrier with a semiconductor material at the interface that improve charge transfer and alleviate the recombination rate by the established built-in electric field. Simultaneously, the metallic nature of graphene or CNTs efficiently collect photogenerated charges from the semiconductor [125,126]. Photogenerated electrons move by the built-in electrical field from conduction band of semiconductor to CNTs in order to balance Fermi levels, while holes exist in the semiconductor share in redox reaction [127]. Padmanabhan et al. [128] prepared a study showing that TiO_2_/graphene is more active for photocatalysis than sole TiO_2_. In the report, the graphene sheet acted similar to an electron acceptor facilitating the transfer and separation of the generated electrons during irradiation, thus reducing the e/h recombination. Carbon quantum dots also led the improvement for photocatalytic reaction with TiO_2_. A CQD/TNT nanocomposite yields more than two times higher production rates both for CO and CH_4_ compared to those of bare TNTs [129]. Another study presented by Morawski et al. [130] stated that combining commercial P25 with carbon spheres then depositing this composite on glass fiber fabric showed high efficiency and selectivity in CO_2_ reduction into CO.

## 5. Strategy IV: Modified TiO_2_ Nanostructures by Hydrogenation

The next strategy applicable for efficient TiO_2_ photocatalysts is to modify its properties. Several approaches have been explored to modify properties of TiO_2_ through conventional doping process. Doping of metal ions (Cr, Sn, Zn, W, etc.) and nonmetallic ions (C, P, I, N etc.) into TiO_2_ to create discrete or midgap energy states within its bandgap has been attempted to enhance its electro- and photo-response [131,132]. However, the doping elements are also known to create recombination center simultaneously [131]. While, nonmetallic ion doping is difficult due to the diverse chemical properties of the dopant ions and the existence of O^2−^ in TiO_2_, Nitrogen is one of the elements that has been extensively doped into TiO_2_ for visible light absorption [133,134].

Another way of modifying the properties of TiO_2_ is hydrogenation that is different from the conventional doping method. The hydrogenation is the way of introducing oxygen vacancy by hydrogen element that results in the incorporation of Ti^3+^ to TiO_2_ [135,136]. Namely, Hydrogenation reduces TiO_2_ through the conversion of Ti^4+^ to Ti^3+^ or other states. Depending on the degree or method of hydrogenation, the colors of the hydrogenated TiO_2_ can be varied: black, blue, or brown [137]. Hydrogenation generally results in surface modifications in few nm and leads to the modification of energy band structure of TiO_2_, forming additional energy states located under the conduction band edge [137]. These modified properties of TiO_2_ by the hydrogenation can contribute to many advantageous characteristics for more efficient photocatalytic performance: enhanced light absorptions and control of bandgap. To implement the hydrogenation to TiO_2_, a number of techniques such as electrochemical reduction, metal reduction, NaBH_4_ reduction, laser ablation, microwave radiation, ultrasonication, ion thermal process, and oxidation have been carried out [138,139]. The characteristics of the modified TiO_2_ are affected by various factors, including not only experiment conditions such as reactants, temperature, concentration and pressure of hydrogen and reaction time but also material conditions such as the surface morphology, defect content, shape, and size [140,141,142]. Here this section provides the examples of hydrogenated TiO_2_ synthesis and characteristics, and investigates its applicability to TiO_2_ nanostructures.

The several approaches are valid for the formation of the hydrogenated TiO_2_ to modify nanotubes. The hydrogenated TiO_2_ is basically a reduced form of TiO_2_ and can have extended light absorption region due to the creation of midgap states. In one of the reports, black TiO_2_ has been synthesized via solvothermal method using ethylenediamine followed with calcination of the nanotubes (NT) at 600 °C in a hydrogen atmosphere [143]. The photoelectron spectra of the surface showed Ti^3+^ and oxygen deficiencies (it is also named as self-doping defects) which contributed in bandgap reduction [143]. In another report, 2D TiO_2_ nanosheets have been synthesized via evaporation-induced self-assembly, followed by solvothermal treatment and ethylenediamine reflux [144]. Further, TiO_2_ nanospheres has been obtained via combining hydrogenation with surfactant-induced solvothermal method [145] to achieve reduced TiO_2_ in a controlled manner. These helped the morphology of TiO_2_ to mitigate aggregation and to have low surface energy [145]. Furthermore, 1D TiO_2_ nanotubes have been fabricated via applying hydrogenation and facile solvothermal method [146]. All these reports indicate that controlled hydrogenation leads to the formation of the reduced TiO_2_ that helps in photocatalytic processes [146].

To obtain decent hydrogenation, the experimental conditions in the synthesis process play a significant role in controlling properties of the materials. It has been found that the reactor materials impact the properties: stainless-steel reactor resulted in black powder, while in quartz reactor blue powder was obtained indicating different extents of reduction [147,148]. The initial powder was prepared via mixing 2 g of TiO_2_ in 50 mL NaOH at 120 °C for 48 h, then washed in water and HCl followed by drying overnight at 110 °C to yield titanate nanotubes [142]. Moreover, hydrogen in the atmosphere can easily reduce TiO_2_, and processing in hydrogen ambient resulted in gray TiO_2_. Depending on the degree of hydrogenation and processing conditions, various colors appear on TiO_2_. In another report, the color of protonated titanates converted into brown through calcination at 500 °C in H_2_ atmosphere (N_2_:5%) for 4 h [142]. Hydrogenation of anatase nanowire microspheres demonstrated high visible light absorption and contained Ti-H and O-H bonds which in turn leads to stabilization of surface disordered layer [142]. In addition, the pressure can be also an important factor for the hydrogenation. The hydrogenation of TiO_2_ was implemented using H_2_ pressure at lower temperature with small amount of Pt. In this hydrogenation, hydrogen flow was directed from platinum to TiO_2_, which is known as an advanced reduction procedure [149].

Hydride processes are also applied for the hydrogenation of TiO_2_. When hydrogenation process leads to reduced TiO_2_, the annealing and thermal conditions may pose safety hazards. To avoid these problems, hydride processing has been proposed, in which, hydrides are used in modest conditions to release molecular hydrogen that works as a safe reductant to obtain black TiO_2_ [150]. Both dry and sol-gel methods can be used in hydride reduction. In the dry method, pristine TiO_2_ and hydride are mixed and annealed in Ar and N_2_ atmosphere [151]. In the sol-gel process, NaBH_4_ is used as the reduction agent. NaBH_4_ is added to a mixture of two solutions that is made of (EtOH/HNO_3_) and (EtOH/titanium tetra butoxide), until the gel is reformed. The gel is calcined in a muffle furnace for 3 h to obtain hydrogenated TiO_2_ nanoparticles [152]. Although a completion of hydrogenation may lead to black TiO_2_, other colors is also obtainable by adjusting the temperature and the reduction duration [153]. The synthesized black TiO_2_ nanoparticles were able to absorb > 80% of the sunlight [145]. Nevertheless, the reaction between CaH_2_ and TiO_2_ may result in TiO_3_ and Magneli phase if process is allowed to continue for 240 h. Table 1 summarize hydrogenated TiO_2_ materials by various techniques, their features fabrication, and performance measure for CO_2_ conversion.

## 6. Strategy V: Single Atom Photocatalysts

In the past few years, single atom catalysts (SACs) have nominated as potent photocatalysts that can be employed in CO_2_ reduction efficiently, owing to their compelling properties. First, SACs have high activity and selectivity caused by their distinctive electron structure and unsaturated coordination sites. Second, they have a notable reduction in metal usage brought by the maximum atom utilization. Third, they possess clear reaction mechanisms endowed by the well-defined active sites. Fourth, they help in understanding and realizing the structure and activity relationship due to their atomic scale structure [172,173]. Theoretically, the valence of a single-atom on a support surface supposed to be zero; but practically the value is different. These atoms are being stabilized depending on the covalent coordination or ionic interaction with the supporting surface atoms, hence it possesses partial charge provided via the metal support interactions. In photocatalytic systems, the photogenerated electrons reduces the stabilized ions into metal ions during the photocatalytic reaction [33]. However, Zhang et al. [174] reported a mass production method of a single atom cobalt to be used in photocatalytic CO_2_ reduction. As well as, Xiong et al. [175] presented a study of CO_2_ reduction over Ni single atoms supported on defect-rich ZrO_2_. This strategy requires further studies to define its influencing factors. It is true that throughout the past decades, physicochemical characteristics and functions of these photocatalysts have been attained. Still, more studies required to tailor the electronic and chemical structures in order to widen its use in photocatalytic applications.

## 7. Strategy VI: Metal Organic Framework

Metal organic frameworks (MOFs) are microporous or mesoporous crystalline solid where the lattice is being formed via linking metallic nodes with rigid organic linkers possessing two or more coordination positions; the metallic nodes comprise of metal cations or clusters of few ions of metals. In addition, they can be called porous coordination polymers (PCPs) attributed to the nature of the interaction between the metallic nodes and the organic linkers. MOFs includes almost all the di-, tri- and tetra-positive ions mentioned in the periodic table. The materials that can be used in structure and binding groups of the organic linkers are abundant, as well. Yet, the most renowned ones are organo-phosphorous compounds, aromatic polycarboxylates and nitrogenated heterocycles [176].

MOFs has shown promising potentials in photocatalysis applications and energy conversion [177]. Titanium-based MOFs (Ti-MOFs) are attractive for practical applications, especially tetravalent cation due to its good redox activity, rigid framework and strong metal-ligand bonding [178]. Ti-MOFs represent an exemplary role in MOF family for their rich content, low toxicity, excellent structural topologies, and fascinating photocatalytic activity [179]. The variety of Ti-MOFs has been expanded by manipulating the synthesis parameters of organic ligands and titanium precursors. Nowadays, Ti-MOF derived materials showed high capability in the fields of energy conversion because of their stability, porosity, and regular component arrangement [177]. For instance, preparing Au/TiO_2_ by pyrolyzed Au/NH_2_-MIL-125 boosted CO_2_ reduction into CH_4_ [180]. It was reported that NH_2_-MIL-125 (Ti) reduced CO_2_ into HCOO^−^ in presence of TEOA as electron donor [181]. Zhang et al. synthesized Cu-NH2-MIL-125 (Ti) that showed improved light absorption and ameliorated the charge separation, supported with an extended stability throughout four photocatalysis cycles [182]. The photocatalytic conversion rate of CdS-MIL-125 (Ti) was enhanced by the improved light absorption and e/h pairs separation [183]. Moreover, the reports showed that coupling narrow band-gap semiconductors with Ti-MOFs boosts the photosensitive impact and enhances light absorption capacity [184]. Yang et al. proposed a study about ternary heterostructured MIL-125/Ag/g-C_3_N_4_ nanocomposites that showed efficient photoreduction in visible light [185]. Many other applications in different fields of renewable energy have received extensive attention due to the promising results via reducing the recombination centers, controlling reactive sites and enhancing the light absorption.

## 8. Conclusions

TiO_2_ has been one of the most investigated materials in photocatalysis and studies are in progress among the scientific community to address the unresolved issues. Crystal facet engineering is an important strategy for optimizing both reactivity and selectivity, Researchers have investigated several fabrication routes to control the crystal facets’ type and density. Studies have been able to synthesize photocatalysts with defined facets and their effects on selectivity of product formation have been investigated both theoretically and experimentally, controlling the ratio of different facets and achieving a dominant orientation still remains a challenge.

The nanostructures of TiO_2_ in its several types and morphologies have been fabricated to explore the novel electronic and the optical properties. Anodization is the most common technique to synthesize vertically aligned nanotubes, however, several other of the 1D and 2D structures have been synthesized using various chemical approaches such as solution growth, hydrothermal and sol-gel process. In particular, designing anatase nanocrystals with the most active facet of (001) helped in scrutinizing catalytic activity in a systematic way.

These morphologies and preparation methods directly control the photocatalytic performance of the synthesized nanomaterials in the form of band gap, electronic structure, light absorption, and surface adsorption. These property enhancements have been explained by the existence of Ti^3+^, oxygen vacancies, active surface area, charge separation and photocatalysis response. Moreover, owing to the reduction of the e-h recombination, the subsequent lifetime enhancement, the charge transfer kinetics is enhanced as well.

Nevertheless, there are still several challenges remain with the TiO_2_ nanostructures to achieve higher and better efficiency in photocatalysis. Taking into consideration the different modification approaches of these structures through the synthesis via different methods, there is no unambiguous approach to achieve all properties in the best possible values and hence the synthesis approaches depend on desired morphology or application. However, more investigations are required to achieve in-depth understating of the structure-property relationships in various types and forms of TiO_2_, and to address the issues such as recombination of photo generated charges and extending the light absorption. The heterojunction approach is expected to play a big role in scaling up and commercially viable technologies. By a suitable selection of the heterojunction component (metal or semiconductor), several drawbacks of TiO_2_ such as narrow light absorption, higher recombination, surface area for molecular adsorption can be addressed. Further, the regeneration of the photocatalysts and slowing the rate of degradation are severe challenges for practical and scalable implementation. Hence, more theoretical and experimental studies are required to reach comprehensive understanding of TiO_2_ nanostructures and further research needed to promote its properties.

## Figures and Tables

**Table 1:** Various techniques for the synthesis of hydrogenated TiO_2_ materials and their features in the synthesis processes.

Catalyst	Treatment	Note	H_2_ Evolution Rate/Removal Efficacy	Reference
Black TiO_2_ nanoparticles	Thermal plasma furnace	The absorption increases promptly and monotonously in visible spectrum, when the wavelength is >400 nm	Visible light: 83%	[154]
Black TiO_2_ nanotubes	Hydrogen plasma method	NaOH solution (10M, 50 mL), to be used in heating 2g of P25 for 12 h, then being washed with water and HCL.	7 µmol h^−1^ cm^−2^	[155]
Black TiO_2_ nanoparticles	Electron beam treatment	Electron-beam-assisted high energy electron used in changing the composition of TiO_2_. Electron beam maximum energy 0.7 MeV. Electron beam maximum power 28 kW	Visible light: 85%	[156]
TiO_2_ nanotubes with black appearance for the proton-implanted layer	Proton implantation	The top of the nanotubes is being modified via high energy proton ion-implantation strategy. Then implanting the substrate with Varian 350D ion implanter. The resulted nanotubes showed high performance in aqueous solution.	UV: 38%	[157]
Defective TiO_2_	Metal reduction	Metals like Zn, Al, Mg are excellent reductants that for being cheap, safe and convenient in comparison with hydrogen.	Solar light: 95%	[142]
Black TiO_2_ and TiO_2_ nanotubes	Aluminum reduction	TiO_2_ and Al are being processed in a dual tube furnace below 0.5 Pa	3.9 mmol g^−1^ h^−1^	[158,159]
Gray TiO_2_ nanowires	Aluminum reduction	Titanate nanowires are being processed in double zone furnace in Al atmosphere for 4 h	Solar light: 95%	[160]
Black brookite TiO_2_ nanoparticles	Aluminum reduction	Brookite TiO_2_ and Al powder are being placed in dual vacuum furnace and heated for 4 h at 300–600 and 800 °C. This process promoted the absorption of visible spectrum and IR of brookite TiO_2_	Solar light: 92%	[159]
Black rutile TiO_2_ Nanoparticles	Molten Aluminum	The sample is being heated at 550–800 °C at a pressure of 6 × 10^−4^ Pa in a vacuum-double-zone furnace. And the results showed enhanced absorption.	932 µmol h^−1^ g^−1^	[161]
Black TiO_2−x_ nanoparticles	Al powder	Al powder and P25 (0.5 g) are being processed in a two-zone vacuum furnace. Then using thermal plasma furnace to apply hydrogen plasma for 5 h	15 mmol h^−1^ g^−1^	[162]
Black TiO_2_-N nanoparticles		The material is being heated in a gas stream of NH_3_-Ar	Solar light: 85%	[162]
Rutile TiO_2_ nanoparticles	Zn reduction	Mixing aqueous TiCl_2_ (1 mL) and isopropanol (30 mL) at 180 °C in existence of Zn powder for 6 h.	1.4 mmol h^−1^ g^−1^	[163]
Black TiO_2_ photocatalyst	Mg reduction	Mixing TiO_2_ with Mg powder resulted black TiO_2_. But Mg and H_2_ resulted in highly stable and active reduced black TiO_2_.	440 µmol h^−1^ g^−1^	[164]
Porous amorphous Vo-TiO_2_	Organic reduction	300-Xe lamp has been used as a light source. The target is aqueous methanol solution (25 vol%, 120 mL) for 8 h in UV and visible light: 5.67 mmol h^−1^ g^−1^ For 14 h, in visible light radiation: 115 µmol h^−1^ g^−1^	Visible light and UV: 5.67 mmol h^−1^ g^−1^	[165]
Ti^3+^ doped TiO_2_	Organic reduction	300 W Xe lamp, aqueous methanol solution (25 vol%, 120 mL), for 4 h in visible light irradiation: 50 μmol h^−1^ g^−1^	Visible light: 115 µmol h^−1^ g^−1^	[166]
Defective TiO_2−x_	Organic reduction	Imidiazole and 2-ethylimidazole	-	[165,166,167]
Gray TiO_2_	Organic reduction	A TiO_2_ precursor exposed to UV for one hour then annealed with hydrochloric acid, and imidazole (1 g) in a muffle furnace at 450 °C	115 µmol h^−1^ g^−1^	[165]
Black defective TiO_2_ nanotubes	Electrochemical reduction	TiO_2_ were synthesized via Ti foil anodization in (4 mA for 5000 s or 80 V for 7200 s). then calcined in air.	Visible light: 72%	[168]
Ti^3+^ self-doped TiO_2−x_ nanoparticles	Chemical oxidation	The light source used is 300 W Xe lamp. Target and concentration are aqueous methanol solution (100 mL, 20%) MB (120 mL, 5 × 10^−4^ mol/L), for 4 h.	250 µmol h^−1^ g^−1^	[169]
Ti^3+^ self-doped rutile TiO_2_	Chemical oxidation	Using solar simulator, MB (30 mL, 10^−5^ M), for 1 h	-	[170]
Ti^3+^ self-doped TiO_2−x_ anatase nanoparticles	Chemical oxidation	Light source: 300 W Xe arc lamp MB (100 mL, 1.5 × 10^−5^ mol/L) aqueous methanol solution (20 vol%) for 30 min	147 µmol h^−1^ g^−1^	[171]

## References

[1] Mardani A., Streimikiene D., Cavallaro F., Loganathan N., Khoshnoudi M. (2017). Carbon dioxide (CO_2_) emissions and economic growth: A systematic review of two decades of research from 1995 to 2017. Sci. Total Environ..

[2] Pipes R., Bhargav A., Manthiram A. (2019). Phenyl Disulfide Additive for Solution-Mediated Carbon Dioxide Utilization in Li−CO_2_ Batteries. Adv. Energy Mater..

[3] Song C., Liu Q., Deng S., Li H., Kitamura Y. (2019). Cryogenicbased CO_2_ capture technologies: State-of-the-art developments and current challenges. Renew. Sustain. Energy Rev..

[4] Duan Y.-X., Meng F.-L., Liu K.-H., Yi S.-S., Li S.-J., Yan J.-M., Jiang Q. (2018). Amorphizing of Cu nanoparticles toward highly efficient and robust electrocatalyst for CO_2_ reduction to liquid fuels with high faradaic efficiencies. Adv. Mater..

[5] Sun Z., Ma T., Tao H., Fan Q., Han B. (2017). Fundamentals and challenges of electrochemical CO_2_ reduction using two-dimensional materials. Chem.

[6] Zhang N., Long R., Gao C., Xiong Y. (2018). Recent progress on advanced design for photoelectrochemical reduction of CO_2_ to fuels. Sci. China Mater..

[7] Yang D., Yu H., He T., Zuo S., Liu X., Yang H., Ni B., Li H., Gu L., Wang D. (2019). Visible-light-switched electron transfer over single porphyrin-metal atom center for highly selective electroreduction of carbon dioxide. Nat. Commun..

[8] Liu X., Kang F., Hu C., Wang L., Xu Z., Zheng D., Gong W., Lu Y., Ma Y., Wang J. (2018). A genetically encoded photosensitizer protein facilitates the rational design of a miniature photocatalytic CO_2_-reducing enzyme. Nat. Chem..

[9] Meng A., Zhang L., Cheng B., Yu J. (2019). TiO_2_−MnO_x_−Pt Hybrid Multiheterojunction Film Photocatalyst with Enhanced Photocatalytic CO_2_-Reduction Activity. ACS Appl. Mater. Interfaces.

[10] Lisovskaya A., Bartels D.M. (2019). Reduction of CO_2_ by hydrated electrons in high temperature water. Radiat. Phys. Chem..

[11] Ferrah D., Haines A.R., Galhenage R.P., Bruce J.P., Babore A.D., Hunt A., Waluyo I., Hemminger J.C. (2019). Wet Chemical Growth and Thermocatalytic Activity of Cu-Based Nanoparticles Supported on TiO_2_ Nanoparticles/HOPG: In Situ Ambient Pressure XPS Study of the CO_2_ Hydrogenation Reaction. ACS Catal..

[12] Huang J., Buonsanti R. (2019). Colloidal nanocrystals as heterogeneous catalysts for electrochemical CO_2_ conversion. Chem. Mater..

[13] Kang M.J., Kim C.W., Pawar A.U., Cha H.G., Ji S., Cai W.-B., Kang Y.S. (2019). Selective Alcohol on Dark Cathode by Photoelectrochemical CO_2_ Valorization and Their in-situ Characterization. ACS Energy Lett..

[14] Ji Y., Luo Y. (2019). Direct Donation of Protons from H_2_O to CO_2_ in Artificial Photosynthesis on the Anatase TiO_2_ (101) Surface. J. Phys. Chem. Commun..

[15] Xu S., Carter E.A. (2019). Theoretical insights into heterogeneous (Photo) electrochemical CO_2_ reduction. Chem. Rev..

[16] Raizada P., Soni V., Kumar A., Singh P., Khan A.A.P., Asiri A.M., Thakur V.K., Nguyen V.-H. (2021). Surface defect engineering of metal oxides photocatalyst for energy application and water treatment. J. Mater..

[17] Wang P., Wang S., Wang H., Wu Z., Wang L. (2018). Recent progress on photo-electrocatalytic reduction of carbon dioxide. Part. Part. Syst. Charact..

[18] Wang L., Chen W., Zhang D., Du Y., Amal R., Qiao S., Wu J., Yin Z. (2019). Surface strategies for catalytic CO_2_ reduction: From two-dimensional materials to nanoclusters to single atoms. Chem. Soc. Rev..

[19] Kong L., Qiao J., Ruan Q., Wang H., Xi X., Zha W., Zhou Z., He W., Zhang W., Sun Z. (2022). A very low charge potential for zinc-air battery promoted by photochemical effect of triazine-based conjugated polymer nanolayer coated TiO_2_. J. Power Sources.

[20] Pawar A.U., Kim C.W., Nguyen-Le M.-T., Kang Y.S. (2019). General review on the components and parameters of photoelectrochemical system for CO_2_ reduction with in situ analysis. ACS Sustain. Chem. Eng..

[21] Inoue T., Fujishima A., Konishi S., Honda K. (1979). Photoelectrocatalytic reduction of carbon dioxide in aqueous suspensions of semiconductor powders. Nature.

[22] Fujishima A., Honda K. (1972). Electrochemical photolysis of water at a semiconductor electrode. Nature.

[23] Tamgadge R.M., Shukla A. (2018). Fluorine-doped anatase for improved supercapacitor electrode. Electrochim. Acta.

[24] Hussain I., Chowdhury A.R., Jaksik J., Grissom G., Touhami A., Ibrahim E.E., Schauer M., Okoli O., Uddin M.J. (2019). Conductive glass free carbon nanotube micro yarn based perovskite solar cells. Appl. Surf. Sci..

[25] Mino L., Ferrari A.M., Lacivita V., Spoto G., Bordiga S., Zecchina A. (2011). CO adsorption on anatase nanocrystals: A combined experimental and periodic DFT study. J. Phys. Chem. C.

[26] Das S., Dhara S. (2021). Chemical Solution Synthesis for Materials Design and Thin Film Device Applications.

[27] Mino L., Zecchina A., Martra G., Rossi A.M., Spoto G. (2016). A surface science approach to TiO_2_ P25 photocatalysis: An in situ FTIR study of phenol photodegradation at controlled water coverages from sub-monolayer to multilayer. Appl. Catal. B Environ..

[28] Li G., Fang K., Ou Y., Yuan W., Yang H., Zhang Z., Wang Y. (2021). Surface study of the reconstructed anatase TiO_2_ (001) surface. Prog. Nat. Sci. Mater. Int..

[29] Liu Y., Du Y.E., Bai Y., An J., Li J., Yang X., Feng Q. (2018). Facile Synthesis of {101},{010} and [111]-Faceted Anatase-TiO_2_ Nanocrystals Derived from Porous Metatitanic Acid H_2_TiO_3_ for Enhanced Photocatalytic Performance. ChemistrySelect.

[30] Mollavali M., Rohani S., Elahifard M., Behjatmanesh-Ardakani R., Nourany M. (2021). Band gap reduction of (Mo+ N) co-doped TiO_2_ nanotube arrays with a significant enhancement in visible light photo-conversion: A combination of experimental and theoretical study. Int. J. Hydrog. Energy.

[31] Grissom G., Jaksik J., McEntee M., Durke E.M., Aishee S.T., Cua M., Okoli O., Touhami A., Moore H.J., Uddin M.J. (2018). Three-dimensional carbon nanotube yarn based solid state solar cells with multiple sensitizers exhibit high energy conversion efficiency. Solar Energy.

[32] Likodimos V. (2018). Photonic crystal-assisted visible light activated TiO_2_ photocatalysis. Appl. Catal. B Environ..

[33] Cravanzola S., Cesano F., Gaziano F., Scarano D. (2017). Sulfur-doped TiO_2_: Structure and surface properties. Catalysts.

[34] Humayun M., Raziq F., Khan A., Luo W. (2018). Modification strategies of TiO_2_ for potential applications in photocatalysis: A critical review. Green Chem. Lett. Rev..

[35] Uddin M.J., Daramola D.E., Velasquez E., Dickens T.J., Yan J., Hammel E., Cesano F., Okoli O.I. (2014). A high efficiency 3D photovoltaic microwire with carbon nanotubes (CNT)-quantum dot (QD) hybrid interface. Phys. Status Solidi (RRL)–Rapid Res. Lett..

[36] Jia S., Li X., Zhang B., Yang J., Zhang S., Li S., Zhang Z. (2019). TiO_2_/CuS heterostructure nanowire array photoanodes toward water oxidation: The role of CuS. Appl. Surf. Sci..

[37] Uddin M.J., Cesano F., Chowdhury A.R., Trad T., Cravanzola S., Martra G., Mino L., Zecchina A., Scarano D. (2020). Surface structure and phase composition of TiO_2_ P25 particles after thermal treatments and HF etching. Front. Mater..

[38] Peng Y.-K., Chou H.-L., Tsang S.C.E. (2018). Differentiating surface titanium chemical states of anatase TiO_2_ functionalized with various groups. Chem. Sci..

[39] Zou Y., Gao G., Wang Z., Shi J.-W., Wang H., Ma D., Fan Z., Chen X., Wang Z., Niu C. (2018). Formation mechanism of rectangular-ambulatory-plane TiO_2_ plates: An insight into the role of hydrofluoric acid. Chem. Commun..

[40] Ma M.J., Li W., Dambournet D. (2017). Solution-Based Synthesis of Nano-Sized TiO_2_ Anatase in Fluorinating Media. Modern Synthesis Processes and Reactivity of Fluorinated Compounds.

[41] Wang A., Wu S., Dong J., Wang R., Wang J., Zhang J., Zhong S., Bai S. (2021). Interfacial facet engineering on the Schottky barrier between plasmonic Au and TiO_2_ in boosting the photocatalytic CO_2_ reduction under ultraviolet and visible light irradiation. Chem. Eng. J..

[42] Dong J., Wang Z., Cao H., Xue J., Liu C., Sun S., Gao C., Zhu X., Bao J. (2021). Independent Cr_2_O_3_ functions as efficient cocatalyst on the crystal facets engineered TiO_2_ for photocatalytic CO_2_ reduction. Appl. Surf. Sci..

[43] Butburee T., Kotchasarn P., Hirunsit P., Sun Z., Tang Q., Khemthong P., Sangkhun W., Thongsuwan W., Kumnorkaew P., Wang H. (2019). New understanding of crystal control and facet selectivity of titanium dioxide ruling photocatalytic performance. J. Mater. Chem. A.

[44] Li X. (2019). Synthesis and Metal-Insulator Transition Properties of Vanadium Dioxide Nanostructures.

[45] Du Y.-E., Niu X., He X., Hou K., Liu H., Zhang C. (2021). Synthesis and photocatalytic activity of TiO_2_/CdS nanocomposites with co-exposed anatase highly reactive facets. Molecules.

[46] Du Y.-E., Niu X., He J., Liu L., Liu Y., Chen C., Yang X., Feng Q. (2020). Hollow square rodlike microtubes composed of anatase nanocuboids with coexposed {100},{010}, and {001} facets for improved photocatalytic performance. ACS Omega.

[47] Wen C.Z., Zhou J.Z., Jiang H.B., Hu Q.H., Qiao S.Z., Yang H.G. (2011). Synthesis of micro-sized titanium dioxide nanosheets wholly exposed with high-energy {001} and {100} facets. Chem. Commun..

[48] Li W. (2015). Sol-Gel Synthesis of TiO_2_ Anatase in a Fluorinated Medium and Its Applications as Negative Electrode for Li+ and Na+ Batteries. Ph.D. Thesis.

[49] Bokare A., Erogbogbo F. (2021). Photocatalysis and Li-Ion Battery Applications of {001} Faceted Anatase TiO_2_-Based Composites. J.

[50] Du D.-J., Du Y.-E., Yue W.-B., Yang X.-J. (2019). Lithium storage performance of {010}-faceted and [111]-faceted anatase TiO_2_ nanocrystals. J. Cent. South Univ..

[51] Maitani M.M., Tateyama A., Boix P.P., Han G., Nitta A., Ohtani B., Mathews N., Wada Y. (2019). Effects of energetics with {001} facet-dominant anatase TiO_2_ scaffold on electron transport in CH3NH3PbI3 perovskite solar cells. Electrochim. Acta.

[52] Liu X., Du G., Li M. (2019). True photoreactivity origin of Ti3+-doped anatase TiO_2_ crystals with respectively dominated exposed {001},{101}, and {100} facets. ACS Omega.

[53] Liu G., Wang T., Zhou W., Meng X., Zhang H., Liu H., Kako T., Ye J. (2015). Crystal-facet-dependent hot-electron transfer in plasmonic-Au/semiconductor heterostructures for efficient solar photocatalysis. J. Mater. Chem. C.

[54] Liu L., Liu Z., Liu A., Gu X., Ge C., Gao F., Dong L. (2014). Engineering the TiO_2_–graphene interface to enhance photocatalytic H_2_ production. ChemSusChem.

[55] Pathakoti K., Manubolu M., Hwang H.-M. (2018). Nanotechnology applications for environmental industry. Handbook of Nanomaterials for Industrial Applications.

[56] Kozak M., Mazierski P., Żebrowska J., Kobylański M., Klimczuk T., Lisowski W., Trykowski G., Nowaczyk G., Zaleska-Medynska A. (2018). Electrochemically obtained TiO_2_/CuxOy nanotube arrays presenting a photocatalytic response in processes of pollutants degradation and bacteria inactivation in aqueous phase. Catalysts.

[57] Qu J., Sha L., Wu C., Zhang Q. (2019). Applications of mechanochemically prepared layered double hydroxides as adsorbents and catalysts: A mini-review. Nanomaterials.

[58] Xue Y., Wu Z., He X., Yang X., Chen X., Gao Z. (2019). Constructing a Z-scheme heterojunction of egg-like core@ shell CdS@ TiO_2_ photocatalyst via a facile reflux method for enhanced photocatalytic performance. Nanomaterials.

[59] Shahvaranfard F. (2022). Modification of Low Dimensional Nanostructured TiO_2_ for Energy Application. Ph.D. Thesis.

[60] Lee K., Mazare A., Schmuki P. (2014). One-dimensional titanium dioxide nanomaterials: Nanotubes. Chem. Rev..

[61] Haider A.J., Jameel Z.N., Al-Hussaini I.H. (2019). Review on: Titanium dioxide applications. Energy Procedia.

[62] Ranjan S., Ramalingam C. (2016). Titanium dioxide nanoparticles induce bacterial membrane rupture by reactive oxygen species generation. Environ. Chem. Lett..

[63] Liu Q., Huang J., Tang H., Yu X., Shen J. (2020). Construction 0D TiO_2_ nanoparticles/2D CoP nanosheets heterojunctions for enhanced photocatalytic H_2_ evolution activity. J. Mater. Sci. Technol..

[64] Tahir M., Amin N.S. (2015). Indium-doped TiO_2_ nanoparticles for photocatalytic CO_2_ reduction with H_2_O vapors to CH_4_. Appl. Catal. B Environ..

[65] Wada K., Ranasinghe C.S.K., Kuriki R., Yamakata A., Ishitani O., Maeda K. (2017). Interfacial manipulation by rutile TiO_2_ nanoparticles to boost CO_2_ reduction into CO on a metal-complex/semiconductor hybrid photocatalyst. ACS Appl. Mater. Interfaces.

[66] Tseng I.-H., Chang W.-C., Wu J.C. (2002). Photoreduction of CO_2_ using sol–gel derived titania and titania-supported copper catalysts. Appl. Catal. B Environ..

[67] Wang Y., Lai Q., Zhang F., Shen X., Fan M., He Y., Ren S. (2014). High efficiency photocatalytic conversion of CO_2_ with H_2_O over Pt/TiO_2_ nanoparticles. RSC Adv..

[68] Liu Y., Lee C.-C., Horn M.W., Lee H. (2021). Toward efficient photocatalysts for light-driven CO_2_ reduction: TiO_2_ nanostructures decorated with perovskite quantum dots. Nano Express.

[69] Xu Y.-F., Yang M.-Z., Chen B.-X., Wang X.-D., Chen H.-Y., Kuang D.-B., Su C.-Y. (2017). A CsPbBr3 perovskite quantum dot/graphene oxide composite for photocatalytic CO_2_ reduction. J. Am. Chem. Soc..

[70] Cheng M., Yang S., Chen R., Zhu X., Liao Q., Huang Y. (2017). Copper-decorated TiO_2_ nanorod thin films in optofluidic planar reactors for efficient photocatalytic reduction of CO_2_. Int. J. Hydrog. Energy.

[71] Devi A.D., Pushpavanam S., Singh N., Verma J., Kaur M.P., Roy S.C. (2022). Enhanced methane yield by photoreduction of CO_2_ at moderate temperature and pressure using Pt coated, graphene oxide wrapped TiO_2_ nanotubes. Results Eng..

[72] Rambabu Y., Kumar U., Singhal N., Kaushal M., Jaiswal M., Jain S.L., Roy S.C. (2019). Photocatalytic reduction of carbon dioxide using graphene oxide wrapped TiO_2_ nanotubes. Appl. Surf. Sci..

[73] Ouyang W., Teng F., He J.H., Fang X. (2019). Enhancing the photoelectric performance of photodetectors based on metal oxide semiconductors by charge-carrier engineering. Adv. Funct. Mater..

[74] Wang X., Wang Z., Jiang X., Tao J., Gong Z., Cheng Y., Zhang M., Yang L., Lv J., He G. (2016). Silver-decorated TiO_2_ nanorod array films with enhanced photoelectrochemical and photocatalytic properties. J. Electrochem. Soc..

[75] Qu J., Lai C. (2013). One-dimensional nanostructures as photoanodes for dye-sensitized solar cells. J. Nanomater..

[76] Subramanian A., Pan Z., Li H., Zhou L., Li W., Qiu Y., Xu Y., Hou Y., Muzi C., Zhang Y. (2017). Synergistic promotion of photoelectrochemical water splitting efficiency of TiO_2_ nanorods using metal-semiconducting nanoparticles. Appl. Surf. Sci..

[77] Jiang L., He J., Yang Y., Mao D., Chen D., Wang W., Chen Y., Sharma V.K., Wang J. (2022). Enhancing visible-light photocatalytic activity of hard-biotemplated TiO_2_: From macrostructural morphology replication to microstructural building units design. J. Alloys Compd..

[78] Ghosh M., Liu J., Chuang S.S., Jana S.C. (2018). Fabrication of hierarchical V_2_O_5_ nanorods on TiO_2_ nanofibers and their enhanced photocatalytic activity under visible light. ChemCatChem.

[79] Attar A.S., Ghamsari M.S., Hajiesmaeilbaigi F., Mirdamadi S., Katagiri K., Koumoto K. (2009). Sol–gel template synthesis and characterization of aligned anatase-TiO_2_ nanorod arrays with different diameter. Mater. Chem. Phys..

[80] Wang W.-N., An W.-J., Ramalingam B., Mukherjee S., Niedzwiedzki D.M., Gangopadhyay S., Biswas P. (2012). Size and structure matter: Enhanced CO_2_ photoreduction efficiency by size-resolved ultrafine Pt nanoparticles on TiO_2_ single crystals. J. Am. Chem. Soc..

[81] Ping G., Wang C., Chen D., Liu S., Huang X., Qin L., Huang Y., Shu K. (2013). Fabrication of self-organized TiO_2_ nanotube arrays for photocatalytic reduction of CO_2_. J. Solid State Electrochem..

[82] Feng X., Sloppy J.D., LaTempa T.J., Paulose M., Komarneni S., Bao N., Grimes C.A. (2011). Synthesis and deposition of ultrafine Pt nanoparticles within high aspect ratio TiO_2_ nanotube arrays: Application to the photocatalytic reduction of carbon dioxide. J. Mater. Chem..

[83] Li X., Liu H., Luo D., Li J., Huang Y., Li H., Fang Y., Xu Y., Zhu L. (2012). Adsorption of CO_2_ on heterostructure CdS (Bi_2_S_3_)/TiO_2_ nanotube photocatalysts and their photocatalytic activities in the reduction of CO_2_ to methanol under visible light irradiation. Chem. Eng. J..

[84] Li Y., Wang C., Song M., Li D., Zhang X., Liu Y. (2019). TiO_2−x_/CoOx photocatalyst sparkles in photothermocatalytic reduction of CO_2_ with H_2_O steam. Appl. Catal. B Environ..

[85] Tahir M., Tahir B., Amin N.A.S. (2015). Gold-nanoparticle-modified TiO_2_ nanowires for plasmon-enhanced photocatalytic CO_2_ reduction with H_2_ under visible light irradiation. Appl. Surf. Sci..

[86] Tahir M., Tahir B., Amin N.A.S., Zakaria Z.Y. (2017). Photo-induced reduction of CO_2_ to CO with hydrogen over plasmonic Ag-NPs/TiO_2_ NWs core/shell hetero-junction under UV and visible light. J. CO_2_ Util..

[87] Low J., Qiu S., Xu D., Jiang C., Cheng B. (2018). Direct evidence and enhancement of surface plasmon resonance effect on Ag-loaded TiO_2_ nanotube arrays for photocatalytic CO_2_ reduction. Appl. Surf. Sci..

[88] Su K.-Y., Chen C.-Y., Wu R.-J. (2019). Preparation of Pd/TiO_2_ nanowires for the photoreduction of CO_2_ into renewable hydrocarbon fuels. J. Taiwan Inst. Chem. Eng..

[89] Song G., Xin F., Yin X. (2015). Photocatalytic reduction of carbon dioxide over ZnFe_2_O_4_/TiO_2_ nanobelts heterostructure in cyclohexanol. J. Colloid Interface Sci..

[90] Razzaq A., Grimes C.A., In S.-I. (2016). Facile fabrication of a noble metal-free photocatalyst: TiO_2_ nanotube arrays covered with reduced graphene oxide. Carbon.

[91] Zhang H. (2015). Ultrathin two-dimensional nanomaterials. ACS Nano.

[92] Madkour L.H. (2019). Carbon Nanomaterials and Two-Dimensional Transition Metal Dichalcogenides (2D TMDCs). Nanoelectronic Materials.

[93] Tan C., Cao X., Wu X.-J., He Q., Yang J., Zhang X., Chen J., Zhao W., Han S., Nam G.-H. (2017). Recent advances in ultrathin two-dimensional nanomaterials. Chem. Rev..

[94] Late D.J., Bhat A., Rout C.S. (2019). Fundamentals and properties of 2D materials in general and sensing applications. Fundamentals and Sensing Applications of 2D Materials.

[95] Chen F., Ma T., Zhang T., Zhang Y., Huang H. (2021). Atomic-level charge separation strategies in semiconductor-based photocatalysts. Adv. Mater..

[96] Liu Y., Zou J., Guo B., Ren Y., Wang Z., Song Y., Yu Y., Wu L. (2020). Selective photocatalytic oxidation of thioanisole on DUT-67 (Zr) mediated by surface coordination. Langmuir.

[97] Sadeghfar F., Zalipour Z., Taghizadeh M., Taghizadeh A., Ghaedi M. (2021). Photodegradation processes. Interface Science and Technology.

[98] Tu W., Zhou Y., Liu Q., Yan S., Bao S., Wang X., Xiao M., Zou Z. (2013). An in situ simultaneous reduction-hydrolysis technique for fabrication of TiO_2_-graphene 2D sandwich-like hybrid nanosheets: Graphene-promoted selectivity of photocatalytic-driven hydrogenation and coupling of CO_2_ into methane and ethane. Adv. Funct. Mater..

[99] Zhou S., Liu Y., Li J., Wang Y., Jiang G., Zhao Z., Wang D., Duan A., Liu J., Wei Y. (2014). Facile in situ synthesis of graphitic carbon nitride (g-C3N4)-N-TiO_2_ heterojunction as an efficient photocatalyst for the selective photoreduction of CO_2_ to CO. Appl. Catal. B Environ..

[100] Crake A., Christoforidis K.C., Godin R., Moss B., Kafizas A., Zafeiratos S., Durrant J.R., Petit C. (2019). Titanium dioxide/carbon nitride nanosheet nanocomposites for gas phase CO_2_ photoreduction under UV-visible irradiation. Appl. Catal. B Environ..

[101] Qamar S., Lei F., Liang L., Gao S., Liu K., Sun Y., Ni W., Xie Y. (2016). Ultrathin TiO_2_ flakes optimizing solar light driven CO_2_ reduction. Nano Energy.

[102] Liu Y., Miao C., Yang P., He Y., Feng J., Li D. (2019). Synergetic promotional effect of oxygen vacancy-rich ultrathin TiO_2_ and photochemical induced highly dispersed Pt for photoreduction of CO_2_ with H_2_O. Appl. Catal. B Environ..

[103] Low J., Zhang L., Tong T., Shen B., Yu J. (2018). TiO_2_/MXene Ti_3_C_2_ composite with excellent photocatalytic CO_2_ reduction activity. J. Catal..

[104] Yuan L., Lu K.-Q., Zhang F., Fu X., Xu Y.-J. (2018). Unveiling the interplay between light-driven CO_2_ photocatalytic reduction and carbonaceous residues decomposition: A case study of Bi_2_WO_6_-TiO_2_ binanosheets. Appl. Catal. B Environ..

[105] Reddy K.M., Manorama S.V., Reddy A.R. (2003). Bandgap studies on anatase titanium dioxide nanoparticles. Mater. Chem. Phys..

[106] Munir S., Shah S.M., Hussain H. (2016). Effect of carrier concentration on the optical band gap of TiO_2_ nanoparticles. Mater. Des..

[107] Wang X., Li Z., Shi J., Yu Y. (2014). One-dimensional titanium dioxide nanomaterials: Nanowires, nanorods, and nanobelts. Chem. Rev..

[108] Lee M., Seo Y., Shin H.S., Jo C., Ryoo R. (2016). Anatase TiO_2_ nanosheets with surface acid sites for Friedel–Crafts alkylation. Microporous Mesoporous Mater..

[109] Xu C., Zhang Z., Zhang S., Si H., Ma S., Fan W., Xiong Z., Liao Q., Sattar A., Kang Z. (2021). Manipulation of perovskite crystallization kinetics via Lewis base additives. Adv. Funct. Mater..

[110] Sheng Y., Wei Z., Miao H., Yao W., Li H., Zhu Y. (2019). Enhanced organic pollutant photodegradation via adsorption/photocatalysis synergy using a 3D g-C_3_N_4_/TiO_2_ free-separation photocatalyst. Chem. Eng. J..

[111] Yang K., Cheng G., Chen R., Zhao K., Liang Y., Li W., Han C. (2022). Simply Coupling TiO_2_ Nanospheres with Cu_2_O Particles to Boost the Photocatalytic Hydrogen Evolution through p–n Heterojunction-Induced Charge Transfer. Energy Technol..

[112] Liu Y., Zheng X., Yang Y., Li J., Liu W., Shen Y., Tian X. (2022). Photocatalytic Hydrogen Evolution Using Ternary-Metal-Sulfide/TiO_2_ Heterojunction Photocatalysts. ChemCatChem.

[113] Guo Q., Huang Y., Xu H., Luo D., Huang F., Gu L., Wei Y., Zhao H., Fan L., Wu J. (2018). The effects of solvent on photocatalytic properties of Bi_2_WO_6_/TiO_2_ heterojunction under visible light irradiation. Solid State Sci..

[114] Shang M., Wang W., Zhang L., Sun S., Wang L., Zhou L. (2009). 3D Bi_2_WO_6_/TiO_2_ hierarchical heterostructure: Controllable synthesis and enhanced visible photocatalytic degradation performances. J. Phys. Chem. C.

[115] Li Q., Zong L., Li C., Yang J. (2014). Reprint of “Photocatalytic reduction of CO_2_ on MgO/TiO_2_ nanotube films”. Appl. Surf. Sci..

[116] Ran H., Fan J., Zhang X., Mao J., Shao G. (2018). Enhanced performances of dye-sensitized solar cells based on Au-TiO_2_ and Ag-TiO_2_ plasmonic hybrid nanocomposites. Appl. Surf. Sci..

[117] Yao G.-Y., Zhao Z.-Y., Liu Q.-L., Dong X.-D., Zhao Q.-M. (2020). Theoretical calculations for localized surface plasmon resonance effects of Cu/TiO_2_ nanosphere: Generation, modulation, and application in photocatalysis. Sol. Energy Mater. Sol. Cells.

[118] Wang Z., Lee H., Chen J., Wu M., Leung D.Y., Grimes C.A., Lu Z., Xu Z., Feng S.-P. (2020). Synergistic effects of Pd–Ag bimetals and g-C3N4 photocatalysts for selective and efficient conversion of gaseous CO_2_. J. Power Sources.

[119] Taghizadeh A., Taghizadeh M., Sabzehmeidani M.M., Sadeghfar F., Ghaedi M. (2021). Electronic structure: From basic principles to photocatalysis. Interface Science and Technology.

[120] Wang R., Shen J., Sun K., Tang H., Liu Q. (2019). Enhancement in photocatalytic activity of CO_2_ reduction to CH_4_ by 0D/2D Au/TiO_2_ plasmon heterojunction. Appl. Surf. Sci..

[121] Saraev A.A., Kurenkova A.Y., Gerasimov E.Y., Kozlova E.A. (2022). Broadening the Action Spectrum of TiO_2_-Based Photocatalysts to Visible Region by Substituting Platinum with Copper. Nanomaterials.

[122] Zhang Z., Wang Z., Cao S.-W., Xue C. (2013). Au/Pt nanoparticle-decorated TiO_2_ nanofibers with plasmon-enhanced photocatalytic activities for solar-to-fuel conversion. J. Phys. Chem. C.

[123] Mankidy B.D., Joseph B., Gupta V.K. (2013). Photo-conversion of CO_2_ using titanium dioxide: Enhancements by plasmonic and co-catalytic nanoparticles. Nanotechnology.

[124] Tayebi M., Kolaei M., Tayyebi A., Masoumi Z., Belbasi Z., Lee B.-K. (2019). Reduced graphene oxide (RGO) on TiO_2_ for an improved photoelectrochemical (PEC) and photocatalytic activity. Sol. Energy.

[125] Mondal A., Prabhakaran A., Gupta S., Subramanian V.R. (2021). Boosting photocatalytic activity using reduced graphene oxide (RGO)/semiconductor nanocomposites: Issues and future scope. ACS Omega.

[126] Shin D.H., Choi S.-H. (2018). Graphene-based semiconductor heterostructures for photodetectors. Micromachines.

[127] Sun P., Zhou S., Yang Y., Liu S., Cao Q., Wang Y., Wågberg T., Hu G. (2022). Artificial chloroplast-like phosphotungstic acid—Iron oxide microbox heterojunctions penetrated by carbon nanotubes for solar photocatalytic degradation of tetracycline antibiotics in wastewater. Adv. Compos. Hybrid Mater..

[128] Padmanabhan N.T., Thomas N., Louis J., Mathew D.T., Ganguly P., John H., Pillai S.C. (2021). Graphene coupled TiO_2_ photocatalysts for environmental applications: A review. Chemosphere.

[129] Zhang J., Xu J., Tao F. (2021). Interface Modification of TiO_2_ Nanotubes by Biomass-Derived Carbon Quantum Dots for Enhanced Photocatalytic Reduction of CO_2_. ACS Appl. Energy Mater..

[130] Morawski A.W., Ćmielewska K., Witkowski K., Kusiak-Nejman E., Pełech I., Staciwa P., Ekiert E., Sibera D., Wanag A., Gano M. (2021). CO_2_ Reduction to Valuable Chemicals on TiO_2_-Carbon Photocatalysts Deposited on Silica Cloth. Catalysts.

[131] Rajaraman T.S., Parikh S.P., Gandhi V.G. (2020). Black TiO_2_: A review of its properties and conflicting trends. Chem. Eng..

[132] Mezzat F., Zaari H., El Kenz A., Benyoussef A. (2021). Effect of metal and non metal doping of TiO_2_ on photocatalytic activities: Ab initio calculations. Opt. Quantum Electron..

[133] Jeon J.P., Kweon D.H., Jang B.J., Ju M.J., Baek J.B. (2020). Enhancing the photocatalytic activity of TiO_2_ catalysts. Adv. Sustain. Syst..

[134] Xu T., Wang M., Wang T. (2019). Effects of N doping on the microstructures and optical properties of TiO_2_. J. Wuhan Univ. Technol. Mater. Sci. Ed..

[135] Yamazaki Y., Toyonaga T., Doshita N., Mori K., Kuwahara Y., Yamazaki S., Yamashita H. (2021). Crystal Facet Engineering and Hydrogen Spillover-Assisted Synthesis of Defective Pt/TiO_2_–x Nanorods with Enhanced Visible Light-Driven Photocatalytic Activity. ACS Appl. Mater. Interfaces.

[136] Chen X., Peng X., Jiang L., Yuan X., Fei J., Zhang W. (2022). Photocatalytic removal of antibiotics by MOF-derived Ti^3+^-and oxygen vacancy-doped anatase/rutile TiO_2_ distributed in a carbon matrix. Chem. Eng. J..

[137] Sorcar S., Hwang Y., Grimes C.A., In S.-I. (2017). Highly enhanced and stable activity of defect-induced titania nanoparticles for solar light-driven CO_2_ reduction into CH4. Mater. Today.

[138] Nas M.S., Calimli M.H. (2021). Recent Development of Nanoparticle by Green-Conventional Methods and Applications for Corrosion and Fuel Cells. Curr. Nanosci..

[139] Behera A. (2022). Nanomaterials. Advanced Materials.

[140] Gupta T., Cho J., Prakash J. (2021). Hydrothermal synthesis of TiO_2_ nanorods: Formation chemistry, growth mechanism, and tailoring of surface properties for photocatalytic activities. Mater. Today Chem..

[141] Wang Y.-H., Rahman K.H., Wu C.-C., Chen K.-C. (2020). A review on the pathways of the improved structural characteristics and photocatalytic performance of titanium dioxide (TiO_2_) thin films fabricated by the magnetron-sputtering technique. Catalysts.

[142] Li Z., Wang S., Wu J., Zhou W. (2022). Recent progress in defective TiO_2_ photocatalysts for energy and environmental applications. Renew. Sustain. Energy Rev..

[143] Zhang X., Hu W., Zhang K., Wang J., Sun B., Li H., Qiao P., Wang L., Zhou W. (2017). Ti^3+^ self-doped black TiO_2_ nanotubes with mesoporous nanosheet architecture as efficient solar-driven hydrogen evolution photocatalysts. ACS Sustain. Chem. Eng..

[144] Wu J., Qiao P., Li H., Xu Y., Yang W., Yang F., Lin K., Pan K., Zhou W. (2020). Engineering surface defects on two-dimensional ultrathin mesoporous anatase TiO_2_ nanosheets for efficient charge separation and exceptional solar-driven photocatalytic hydrogen evolution. Mater. Chem..

[145] Li Z., Wang S., Xie Y., Yang W., Tao B., Lu J., Wu J., Qu Y., Zhou W. (2021). Surface defects induced charge imbalance for boosting charge separation and solar-driven photocatalytic hydrogen evolution. Colloid Interface Sci..

[146] Yang W.T., Li M., Pan K., Guo L.P., Wu J.X., Li Z.J., Yang F., Lin K., Zhou W. (2021). Surface engineering of mesoporous anatase titanium dioxide nanotubes for rapid spatial charge separation on horizontal-Vertical dimensions and efficient solar-driven photocatalytic hydrogen evolution. Colloid Interface Sci..

[147] Liu X., Zhu G., Wang X., Yuan X., Lin T., Huang F. (2016). Progress in black titania: A new material for advanced photocatalysis. Adv. Energy Mater..

[148] Shoneye A., Sen Chang J., Chong M.N., Tang J. (2022). Recent progress in photocatalytic degradation of chlorinated phenols and reduction of heavy metal ions in water by TiO_2_-based catalysts. Int. Mater. Rev..

[149] Janczarek M., Kowalska E. (2021). Defective dopant-free TiO_2_ as an efficient visible light-active photocatalyst. Catalysts.

[150] Dobrosielska M., Zieliński M., Frydrych M., Pietrowski M., Marciniak P., Martyła A., Sztorch B., Przekop R.E. (2021). Sol–Gel Approach for Design of Pt/Al_2_O_3_-TiO_2_ System—Synthesis and Catalytic Tests. Ceramics.

[151] Lluna-Galán C., Izquierdo-Aranda L., Adam R., Cabrero-Antonino J.R. (2021). Catalytic Reductive Alcohol Etherifications with Carbonyl-Based Compounds or CO_2_ and Related Transformations for the Synthesis of Ether Derivatives. ChemSusChem.

[152] Tian J., Hu X., Yang H., Zhou Y., Cui H., Liu H. (2016). High yield production of reduced TiO_2_ with enhanced photocatalytic activity. Appl. Surf. Sci..

[153] Fang W., Xing M., Zhang J. (2014). A new approach to prepare Ti^3+^ self-doped TiO_2_ via NaBH4 reduction and hydrochloric acid treatment. Appl. Catal. B Environ..

[154] Tan H., Zhao Z., Niu M., Mao C., Cao D., Cheng D., Feng P., Sun Z. (2014). A facile and versatile method for preparation of colored TiO_2_ with enhanced solar-driven photocatalytic activity. Nanoscale.

[155] Wang Z., Yang C., Lin T., Yin H., Chen P., Wan D., Xu F., Huang F., Lin J., Xie X. (2013). H-doped black titania with very high solar absorption and excellent photocatalysis enhanced by localized surface plasmon resonance. Adv. Funct Mater..

[156] Teng F., Li M., Gao C., Zhang G., Zhang P., Wang Y., Chen L., Xie E. (2014). Preparation of black TiO_2_ by hydrogen plasma assisted chemical vapor deposition and its photocatalytic activity. Appl. Catal. B Environ..

[157] Khan M.M., Ansari S.A., Pradhan D., Ansari M.O., Han D.H., Lee J., Cho M.H. (2014). Band gap engineered TiO_2_ nanoparticles for visible light induced photoelectrochemical and photocatalytic studies. Mater. Chem..

[158] Liu N., Haublein V., Zhou X.M., Venkatesan U., Hartmann M., Mackovic M., Nakajima T., Spiecker E., Osvet A., Frey L. (2015). “Black” TiO_2_ nanotubes formed by high-energy proton implantation show noble-metal-co-catalyst free photocatalytic H_2_-evolution. Nano Lett..

[159] Wang Z., Yang C., Lin T., Yin H., Chen P., Wan D., Xu F., Huang F., Lin J., Xie X. (2013). Visible-light photocatalytic, solar thermal and photoelectrochemical properties of aluminium-reduced black titania. Energy Environ. Sci..

[160] Zhu G., Lin T., Lü X., Zhao W., Yang C., Wang Z., Yin H., Liu Z., Huang F., Lin J. (2013). Black brookite titania with high solar absorption and excellent photocatalytic performance. Mater. Chem..

[161] Yin H., Lin T., Yang C., Wang Z., Zhu G., Xu T., Xie X., Huang F., Jiang M. (2013). Gray TiO_2_ nanowires synthesized by aluminum-mediated reduction and their excellent photocatalytic activity for water cleaning. Chem. Eur. J..

[162] Yang C., Wang Z., Lin T., Yin H., Lü X., Wan D., Xu T., Zheng C., Lin J., Huang F. (2013). Core-shell nanostructured “black” rutile titania as excellent catalyst for hydrogen production enhanced by sulfur doping. Am. Chem. Soc..

[163] Lin T., Yang C., Wang Z., Yin H., Lü X., Huang F., Lin J., Xie X., Jiang M. (2014). Effective nonmetal incorporation in black titania with enhanced solar energy utilization. Energy Environ. Sci..

[164] Zhao Z., Tan H., Zhao H., Lv Y., Zhou L.-J., Song Y., Sun Z. (2014). Reduced TiO_2_ rutile nanorods with well-defined facets and their visible-light photocatalytic activity. Chem. Commun..

[165] Sinhamahapatra A., Jeon J.-P., Yu J.-S. (2015). A new approach to prepare highly active and stable black titania for visible light-assisted hydrogen production. Energy Env. Sci..

[166] Zou X., Liu J., Su J., Zuo F., Chen J., Feng P. (2013). Facile synthesis of thermal- and photostable titania with paramagnetic oxygen vacancies for visible-light photocatalysis. Chem. Eur. J..

[167] Zuo F., Wang L., Wu T., Zhang Z.Y., Borchardt D., Feng P.Y. (2010). Self-doped Ti^3+^ enhanced photocatalyst for hydrogen production under visible light. Am. Chem. Soc..

[168] Chen X., Liu L., Huang F. (2015). Black titanium dioxide (TiO_2_) nanomaterials. Chem. Soc. Rev..

[169] Li H., Chen Z., Tsang C.K., Li Z., Ran X., Lee C., Nie B., Zheng L., Hung T., Lu J. (2014). Electrochemical doping of anatase TiO_2_ in organic electrolytes for high-performance supercapacitors and photocatalyst. Mater. Chem..

[170] Liu X., Gao S., Xu H., Lou Z., Wang W., Huang B., Dai Y. (2013). Green synthetic approach for Ti^3+^ self-doped TiO_2−x_ nanoparticles with efficient visible light photocatalytic activity. Nanoscale.

[171] Grabstanowicz L.R., Gao S., Li T., Rickard R.M., Rajh T., Liu D.J., Xu T. (2013). Facile oxidative conversion of TiH_2_ to high-concentration Ti3+-self-doped rutile TiO_2_ with visible-light photoactivity. Inorg. Chem..

[172] Liu X., Xu H., Grabstanowicz L.R., Gao S., Lou Z., Wang W., Huang B., Dai Y., Xu T. (2014). Ti^3+^ self-doped TiO_2−x_ anatase nanoparticles via oxidation of TiH_2_ in H_2_O_2_. Catal. Today.

[173] Li X., Yang X., Huang Y., Zhang T., Liu B. (2019). Supported noble-metal single atoms for heterogeneous catalysis. Adv. Mater..

[174] Huang P., Huang J., Pantovich S.A., Carl A.D., Fenton T.G., Caputo C.A., Grimm R.L., Frenkel A.I., Li G. (2018). Selective CO_2_ reduction catalyzed by single cobalt sites on carbon nitride under visible-light irradiation. J. Am. Chem. Soc..

[175] Zhang P., Zhan X., Xu L., Fu X., Zheng T., Yang X., Xu Q., Wang D., Qi D., Sun T. (2021). Mass production of a single-atom cobalt photocatalyst for high-performance visible-light photocatalytic CO_2_ reduction. J. Mater. Chem. A.

[176] Xiong X., Mao C., Yang Z., Zhang Q., Waterhouse G.I., Gu L., Zhang T. (2020). Photocatalytic CO_2_ reduction to CO over Ni single atoms supported on defect-rich zirconia. Adv. Energy Mater..

[177] Dhakshinamoorthy A., Li Z., Garcia H. (2018). Catalysis and photocatalysis by metal organic frameworks. Chem. Soc. Rev..

[178] Chen X., Peng X., Jiang L., Yuan X., Yu H., Wang H., Zhang J., Xia Q. (2020). Recent advances in titanium metal–organic frameworks and their derived materials: Features, fabrication, and photocatalytic applications. Chem. Eng. J..

[179] Yang S., Peng L., Bulut S., Queen W.L. (2019). Recent Advances of MOFs and MOF-Derived Materials in Thermally Driven Organic Transformations. Chem. A Eur. J..

[180] Zhu J., Li P.-Z., Guo W., Zhao Y., Zou R. (2018). Titanium-based metal–organic frameworks for photocatalytic applications. Coord. Chem. Rev..

[181] Khaletskaya K., Pougin A., Medishetty R., Rosler C., Wiktor C., Strunk J., Fischer R.A. (2015). Fabrication of gold/titania photocatalyst for CO_2_ reduction based on pyrolytic conversion of the metal–organic framework NH2-MIL-125 (Ti) loaded with gold nanoparticles. Chem. Mater..

[182] Bueken B., Vermoortele F., Vanpoucke D.E., Reinsch H., Tsou C.C., Valvekens P., De Baerdemaeker T., Ameloot R., Kirschhock C.E., Van Speybroeck V. (2015). A Flexible Photoactive Titanium Metal–Organic Framework Based on a [TiIV3 (μ3-O)(O) 2 (COO) 6] Cluster. Angew. Chem. Int. Ed..

[183] Ao D., Zhang J., Liu H. (2018). Visible-light-driven photocatalytic degradation of pollutants over Cu-doped NH2-MIL-125 (Ti). J. Photochem. Photobiol. A Chem..

[184] Rahmani A., Emrooz H.B.M., Abedi S., Morsali A. (2018). Synthesis and characterization of CdS/MIL-125 (Ti) as a photocatalyst for water splitting. Mater. Sci. Semicond. Processing.

[185] An X., Jimmy C.Y., Wang F., Li C., Li Y. (2013). One-pot synthesis of In2S3 nanosheets/graphene composites with enhanced visible-light photocatalytic activity. Appl. Catal. B Environ..

